# Intra-articular opening osteotomy combined with lateral ligament reconstruction for varus ankle arthritis

**DOI:** 10.1186/s13018-020-02143-1

**Published:** 2021-01-06

**Authors:** Yang Xu, Xing-chen Li, Chang-jun Guo, Xiang-yang Xu

**Affiliations:** 1 Ruijin Hospital, Shanghai Jiao Tong University School of Medicine, Shanghai, China; 2 Ruijin Hospital North, Shanghai Jiao Tong University School of Medicine, Shanghai, China

**Keywords:** Ankle arthritis, Osteotomy, lateral ligament reconstruction

## Abstract

**Background:**

Takakura 3B ankle arthritis is featured as obliteration of ankle space with subchondral bone contact. Among these patients, some have medial distal tibial platform erosion. It is hard to treat this kind of patients. The purpose of this study was to evaluate the therapeutic outcomes of intra-articular opening osteotomy combined with lateral ligament reconstruction for Takakura 3B ankle arthritis with medial distal tibial platform erosion.

**Methods:**

From September 2009 to May 2016, 17 patients with Takakura 3B ankle arthritis were reviewed, including 3 male and 14 female patients. All underwent the operation of intra-articular opening osteotomy combined with lateral ligament reconstruction. All patients were available for analysis. The main outcome measurements included TT angle, AOFAS score, VAS score, SF-36 scale, and AOS scale.

**Results:**

All patients were followed for a mean follow-up of 87.2 months (range, 49 to 129 months). The VAS scale improved from 5.5 ± 1.6 to 2.3 ± 1.9. The mean AOFAS score improved from 47.7 ± 15.7 to 75.8 ± 12.0. The SF-36 scale improved from 41.6 ± 14.0 to 67.7 ± 14.6. The AOS improved from 60.9 ± 13.9 to 28.2 ± 17.7. The TT angle improved from 14.3 ± 5.0° to 5.3 ± 4.0°. The TAS and TLS changed from 83.4 ± 2.6° and 77.5 ± 2.3° to 90.7 ± 2.3° and 78.6 ± 2.2°. However, the LTAS was not corrected significantly.

**Conclusion:**

Intra-articular opening osteotomy combined with lateral ligament reconstruction is an effective method to treat varus ankle arthritis with medial distal tibial platform erosion.

## Background

According to Takakura and Tanaka, varus ankle arthritis was classified into four types: stage 1, early sclerosis and formation of osteophytes without changing of ankle joint space; stage 2, narrowing of medial joint space without subchondral bone contact; stage 3, obliteration of ankle space with subchondral bone contact; and stage 4, varus ankle joint with complete bone contact. Stage 3 was further classified into stages 3A and 3B. Stage 3B ankle arthritis was defined as obliteration of ankle space extended to the roof of the dome of the talus with subchondral bone contact [1, 2].

For young adults and those who do not want to sacrifice the native ankle joint, joint-sparing methods instead of total ankle replacement or ankle arthrodesis are very important. Supramalleolar osteotomy is an effective joint-sparing surgical treatment for varus-type ankle arthritis, especially those with small TAS angles [1–7].

In our practice, we do notice positive outcomes after supramalleolar osteotomy for stage 3B ankle arthritis [8]. However, in some cases, the varus talus repeatedly abrades the medial distal tibial platform after long walking. The medial tibial platform is eroded but the lateral tibial surface angle (LTAS) is normal. Previous studies reported a new kind of technique known as intra-articular opening medial tibial wedge osteotomy with good results [9, 10]. However, stage 3B ankle was considered not suitable for this osteotomy. In Myerson’s study, 4 patients had a type 3B deformity. Two of them underwent ankle arthrodesis and two underwent ankle replacement after the intra-articular opening osteotomy [9].

The purpose of this study was to evaluate the therapeutic outcomes of intra-articular opening osteotomy combined with lateral ligament reconstruction for Takakura 3B ankle arthritis with medial distal tibial platform erosion.

## Materials and methods

The current study was approved by our institutional review board. From September 2009 to May 2016, 17 patients (17 ankles, 7 left and 10 right) with Takakura 3B ankle arthritis were reviewed, including 3 male and 14 female patients. All underwent the operation of intra-articular opening osteotomy combined with lateral ligament reconstruction. The mean age was 52.35 ± 8.05 years. The inclusion criteria were (1) Takakura stage 3B ankle arthritis, (2) medial distal tibial platform erosion, (3) normal lateral tibial surface, and (4) painful ankle arthritis undergoing at least 1-year conservative treatment.

The exclusion criteria were (1) end-stage ankle arthritis, (2) patients with neuropathic arthropathy or rheumatoid arthritis, (3) patients with regional infection around the ankle joint or had other ankle surgeries, and (4) patients with severe osteoporosis or large bone loss.

**Fig. 1:**
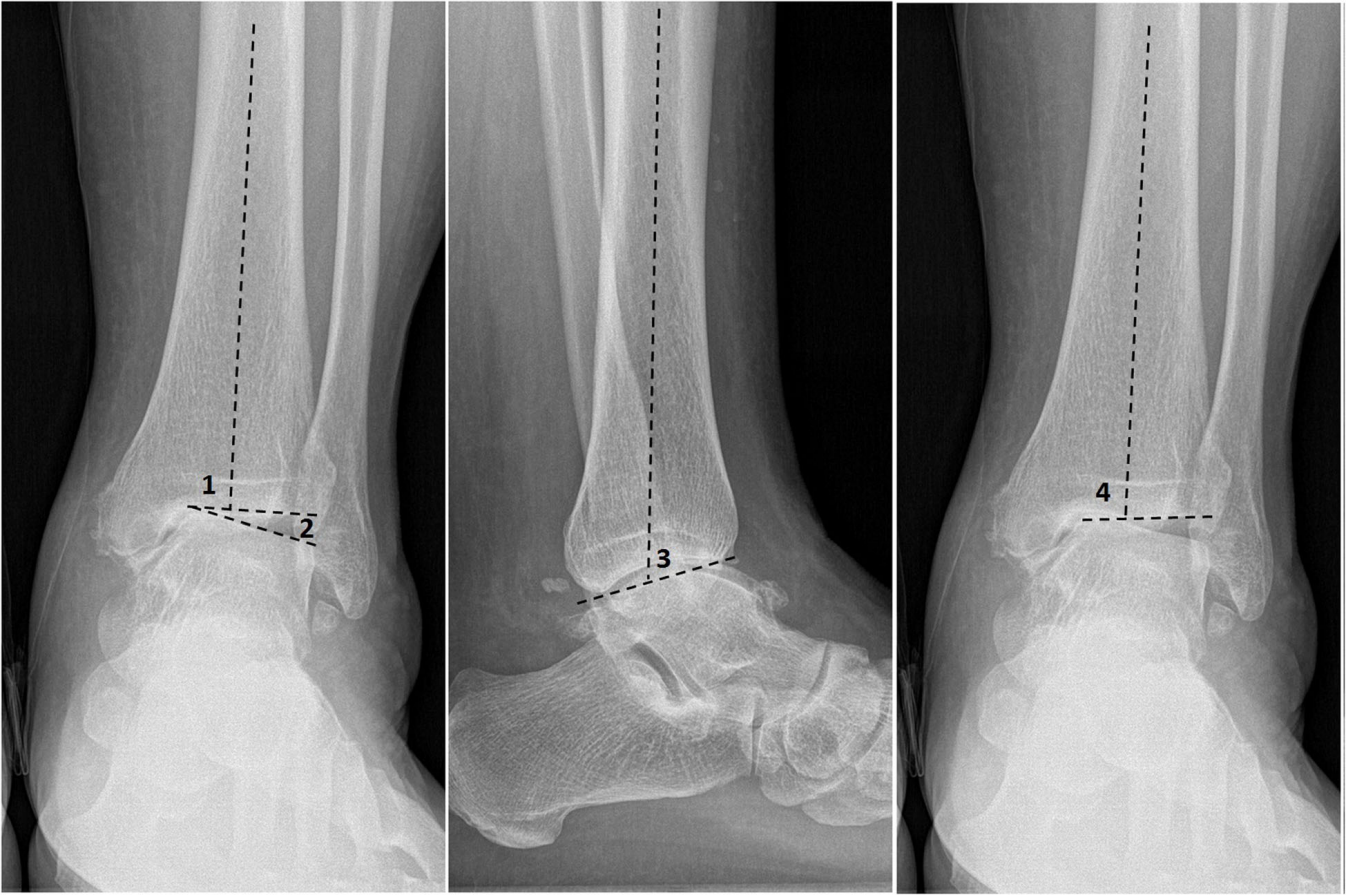
(1) Tibial articular surface angle (TAS). (2) Talar tilt angle (TT). (3) Tibial lateral surface angle (TLS). (4) Lateral tibial articular surface angle (LTAS)

Weightbearing X-rays of ankle joint were performed for every patient preoperatively and postoperatively, including anteroposterior ankle views (AP) and lateral ankle views. In this study, we defined the lateral tibial articular surface angle (LTAS) as the angle between the axis of the tibia and the lateral tibial surface. The talar tilt angle (TT) was regarded as the angle between the distal articular surface of the tibia and the upper surface of the talus. The general tibial articular surface angle (TAS) was defined as the angle between the axis of the tibia and the whole tibial surface. The whole tibial surface was a line drawn from the medial apex of the mortise to the lateral apex of the mortise. The tibial lateral surface angle (TLS) is designated as the angle between the axis of the tibia and the surface of the distal tibia in the lateral view. The hindfoot alignment view, which was recommended by Saltzman and el-Khoury, was also performed to evaluate hindfoot alignment condition [11]. The tibial axis on anteroposterior and lateral radiographs was drawn by connecting the midpoint between the cortex at 13 cm and 8 cm proximal to the joint line. We routinely reviewed ankle MRI scans for every patient to evaluate the cartilage condition. We used the American Orthopaedic Foot & Ankle Society Ankle-Hindfoot Score (AOFAS-AH), the visual analogue scale (VAS), the Short From-36 scale (SF-36), and the Ankle Osteoarthritis scale (AOS) to determine the functional outcome of patients at the final follow-up (Fig. 1).

### Surgical technique

In this study, all ankle arthritis was varus type. The osteotomy approach was through a medial longitudinal incision. A K-wire was placed as a guide wire of osteotomy. Two or three K-wires were placed parallel to the ankle joint surface portion of the tibial platform within the subchondral bone just under the articular cartilage at the apex of the plafond angulation. They help the prevention of destruction of the talus cartilage from the saw blade during the osteotomy. It also acted as a hinge during deformity correction. Then the intra-articular osteotomy was performed. The intraoperation fluoroscopic assessment was performed to evaluate whether the tibial articular surface was normal. If the varus talus could not return to normal, then a release procedure of medial ankle ligament including the superficial and deep deltoid ligament was performed. Debridement of osteophytes was performed if there was impingement around the ankle joint such as the anterior distal tibial osteophyte to improve ankle motion through the medial incision and lateral incision. A wedged allograft was shaped and inserted into the osteotomy site and was fixed by a locking plate. The ankle joint was flexible, and we fixed it into a neutral position with 1–2 K-wires, which were removed 3–4 weeks postoperatively. In this position, the medial ligaments were sutured and lateral ligaments were reconstructed.

**Fig. 2:**
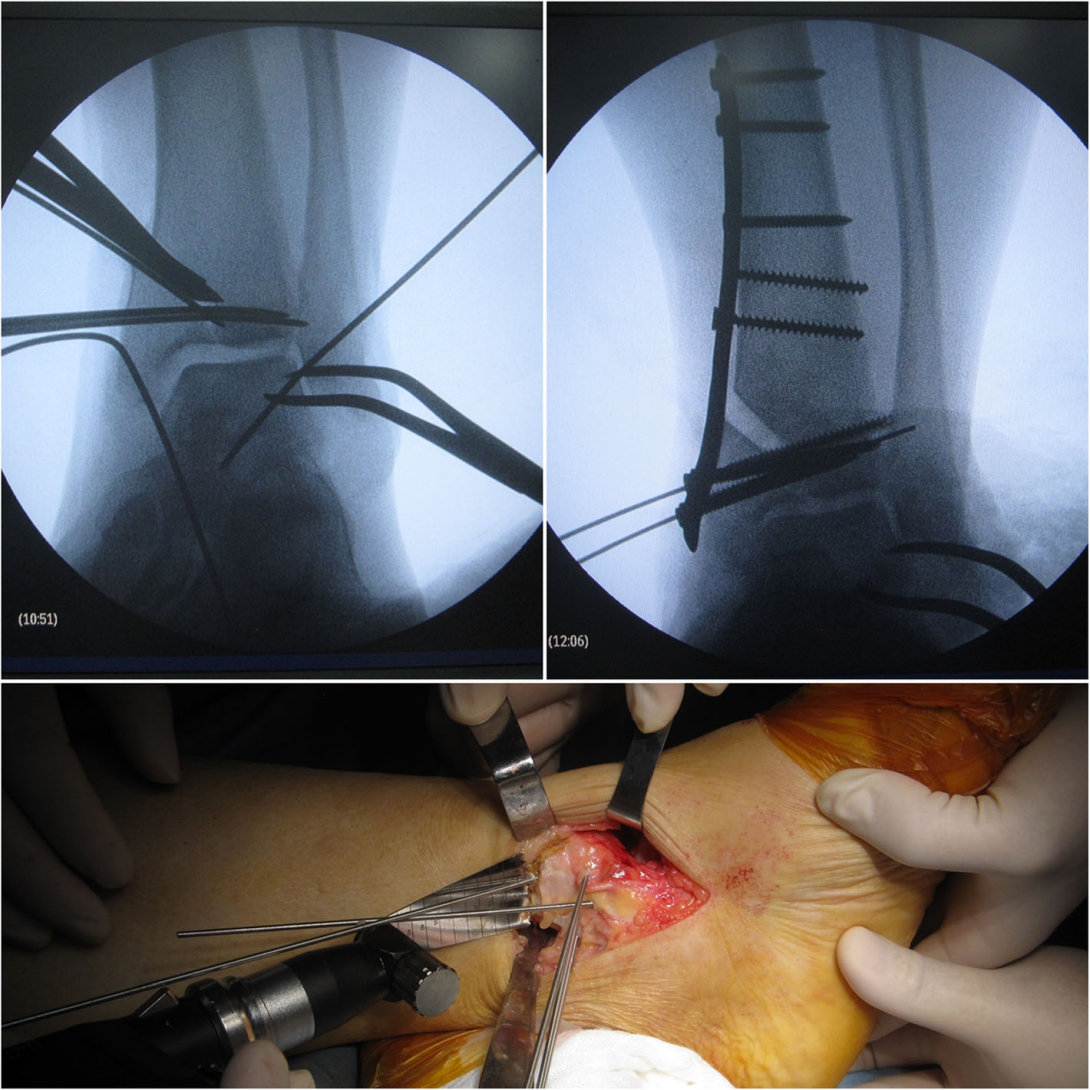
Intraoperative photos showing the osteotomy

Lateral ligament reconstruction was performed via a minimally invasive method [12]. Fifteen patients used allograft, 2 patients autograft. Two guide wires were introduced at the distal end of the fibula, and then a tunnel was made. A hole was made at the lateral side of the talar neck. These were all through the lateral incision. A small incision was made at the lateral side of the middle portion of the calcaneus and a hole was made. The tendon graft was introduced through the tunnel, and the two ends of the tendon were then passed above the bone surface to the holes made at the talar neck and calcaneus fixed by two biodegradable inference screws. The ligament procedures were performed after fixation of osteotomy (Fig. 2).

A cast was used for 6 weeks. Part weight bearing was allowed 6 weeks postoperatively with the cast. Full weight bearing was allowed 2 to 3 months postoperatively. Patients came to the hospital 2 weeks, 6 weeks, 3 months, and 1 year postoperatively. All the patients were followed up annually.

### Statistical methods

All analyses were performed with the SAS software version 8.1 (SAS Institute Inc, Cary, NC). The results were given as means and standard deviation. The paired *t* test was used for assessing differences between preoperative and postoperative measurements. A *p* value less than 0.05 was considered to indicate statistical significance.

## Results

All patients were followed. Patients were followed for a mean follow-up of 87.2 months (range, 49 to 129 months). There was no loss of follow-up. The VAS scale improved from 5.5 ± 1.6 to 2.3 ± 1.9. The mean AOFAS score improved from 47.7 ± 15.7 to 75.8 ± 12.0. The SF-36 scale improved from 41.6 ± 14.0 to 67.7 ± 14.6. The AOS improved from 60.9 ± 13.9 to 28.2 ± 17.7. The TT angle improved from 14.3 ± 5.0° to 5.3 ± 4.0°. The TAS and TLS changed from 83.4 ± 2.6° and 77.5 ± 2.3° to 90.7 ± 2.3° and 78.6 ± 2.2°. However, the LTAS was not corrected significantly.

In this study, 2 patients rated their outcomes as “excellent” and 7 patients rated their outcomes as “good.” Four patients rated their results as “fair.” On the other hand, a “poor” outcome was observed in 4 patients because of the consistent discomfort. However, none of the patients underwent ankle joint arthroplasty or arthrodesis because of the cost involved or other reasons.

**Fig. 3:**
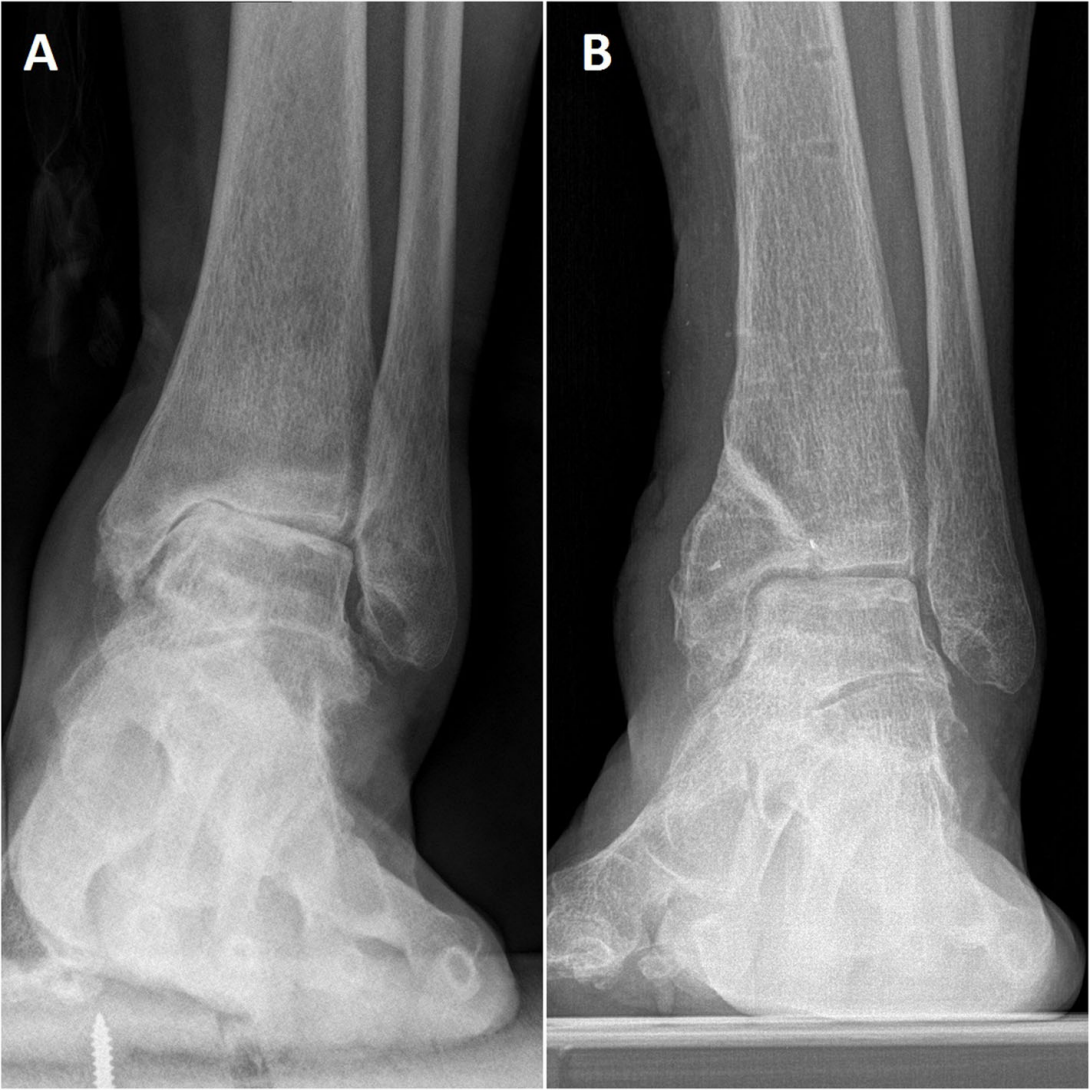
A 55-year-old woman with ankle arthritis before (**a**) and after (**b**) the surgery

**Fig. 4:**
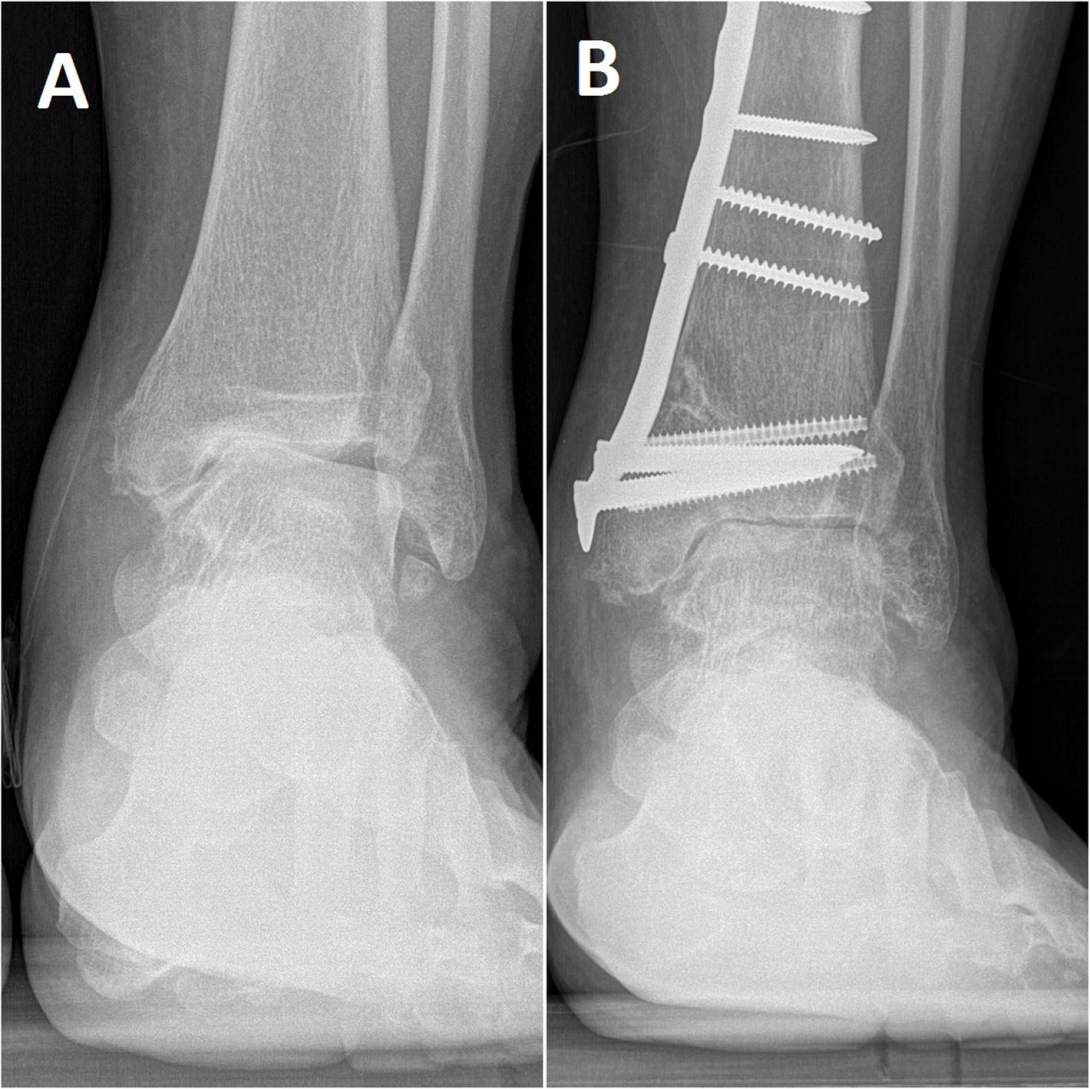
A 49-year-old man with ankle arthritis before (**a**) and after (**b**) the surgery

Ten patients underwent calcaneal osteotomy. Thirteen patients recalled an old ankle sprain history. All of them had a long-term ankle sprain history (5–38 years). One patient had a lateral malleolar fracture history. Three patients had no incentive (Figs. 3 and 4) (Tables 1 and 2).

**Table 1:** Summarized demographic data

Parameter	Data
Number of ankles, *n*	17
Male to female, *n* (%)	3:14
Side (left:right), *n* (%)	7:10
Age at surgery (years)	52.35 ± 8.05
History (%)	
Ankle sprain	13 (76.47%)
Ankle fracture	1 (5.9%)
None	3 (17.65%)

**Table 2:** Radiographic and functional outcomes

	Preop	Postop	*P*
VAS	5.5 ± 1.6	2.3 ± 1.9	< 0.001
AOFAS	47.7 ± 15.7	75.8 ± 12.0	< 0.001
SF-36	41.6 ± 14.0	67.7 ± 14.6	< 0.001
AOS	60.9 ± 13.9	28.2 ± 17.7	< 0.001
TT (°)	14.3 ± 5.0	5.3 ± 4.0	< 0.001
TAS (°)	83.4 ± 2.6	90.7 ± 2.3	< 0.001
TLS (°)	77.5 ± 2.3	78.6 ± 2.2	0.0147
LTAS (°)	91.2 ± 2.9	91.3 ± 3.0	0.55

## Discussion

The causes of varus-type ankle arthritis still remain unknown. In other studies, usually, ankle arthritis develops secondary to trauma [4, 7, 13]. However, in this study, more patients recalled an old ankle sprain history. We thought that maybe it was because of the weak lateral ligament resulted in varus-type ankle arthritis.

Takakura and Tanaka divided varus ankle arthritis into 4 stages, stages 1, 2, 3 (3A, 3B), and 4 [1, 2]. Did stage 3B ankle arthritis develop directly from stage 3A? This is controversial. We do not think all of the stage 3B ankle arthritis were evolved from stage 3A ankle arthritis. When the talus invert in ankle mortise, with long time of walking, the varus talus touches the tibia surface and abrades it gradually. So, maybe the stage 3B ankle arthritis developed directly from stage 2. In this kind of patients, the LTAS is usually normal, while the TAS angle is small. We thought that intra-articular opening osteotomy was helpful for 3B ankle arthritis with medial distal tibial platform erosion and the results bear this out.

One of the causes of varus-type ankle arthritis was chronic ankle instability [14, 15]. In this study, all patients underwent lateral ligament reconstruction. Most of the patients in our study recalled repeated ankle sprain histories. The weak lateral ligament resulted in a chronically unstable varus ankle. The medially driven talus causes chronic pressure to the medial malleolus, which makes medial malleolus no longer vertical [9]. Although some patients in this study had no ankle sprain histories, there were still ankle instabilities after the release of ligaments around the ankle joint and debridement of osteophytes. When the talus could return to normal during operation, we think lateral ligament reconstruction was very important to keep the talus in this position.

For varus ankle arthritis, especially Takakura stage 3 or 4 ankle arthritis, previous studies have reported good results of ankle arthrodesis or ankle replacement [16–20]. However, ankle arthrodesis is a joint sacrifice method to treat ankle arthritis, which restricts ankle movement. In this study, the mean age of patients was 52.35 ± 8.05 years. The patients are relatively young, so ankle arthroplasty may not be suitable for them. These patients were still very positive and want to keep their native ankle joint. Osteotomy provided the possibility to preserve their native ankle joint.

Previous studies reported that it was contraindication if the varus ankle was rigid and could not be corrected to normal under fluoroscopic examination before the surgery [9, 21]. However, in our study, we concern more about whether the varus ankle could return to normal intraoperatively or not. We performed a thorough release of ligaments and capsule around the ankle joint if the varus deformity could not be corrected after osteotomy. After the release and debridement procedure, the ankle joint was flexible and we fixed it into a neutral position with 1–2 K-wires, which were removed 3–4 weeks postoperatively. In this position, the medial ligaments were sutured and lateral ligaments were reconstructed. We think that whether the varus ankle could return to normal during operation matters more than preoperative condition. We care more about the joint cartilage than preoperative talar tilt angle.

We think the most important aspect of this kind of procedure is that there is enough residual articular cartilage. Ankle joints with more than 50% residual articular cartilage tend to get better prognosis than those that are not. So, we did an osteotomy procedure for this kind of ankle arthritis. We do not perform osteotomy surgery for stage 4 ankle arthritis. Joint cartilage of stage 4 ankle arthritis was always extensively destructed. For this kind of patients, even though realignment of the ankle joint may alleviate symptoms, joint damage will progress soon.

## Conclusion

For Takakura 3B ankle arthritis with medial distal tibial erosion, intra-articular opening osteotomy combined with lateral ligament reconstruction is an effective method to treat this kind of varus ankle arthritis.

## Data Availability

All data and materials were in full compliance with the journal’s policy.

## Abbreviations

AOFAS: The American Orthopaedic Foot and Ankle Society Ankle-Hindfoot scores
SF-36: The Short Form (36) Health Survey
VAS: Visual analogue scale
AOS: Ankle Osteoarthritis scale
TT: Talar tilt angle
TAS: Tibial articular surface angle
TLS: Tibial lateral surface angle
LTAS: Lateral tibial articular surface angle
